# Supplementary material for: Specified-duration shapers for suppressing residual vibrations
Source: PLoS One. 2022 Nov 28;17(11):e0276669. doi: 10.1371/journal.pone.0276669 (PMC9704665; doi:10.1371/journal.pone.0276669)
Supplement: Attachment — Submitted filename: Response to Reviewers.docx [file pone.0276669.s001.docx]

**Response to Reviewer #1**

Reviewer #1: I read your paper with interest and enjoyed it.

I have two major concerns with the paper. First of all, I do not think that you have given a clear description of the problem you are trying to solve. You state (in lines 70-73) that "In this paper, a novel specified-duration (SD) shaper that satisfies arbitrarily specified shaping time is proposed. The SD shaper is an input shaper that is designed to remove residual vibration during a given specified shaping time. A systematic method is presented to design the SD shaper with an arbitrarily specified shaping time by using impulse vectors." You do not state that you impose any constraints on the sensitivity to changes in the parameters that describe the system. In the body of the paper, as you increase the "dimensionless shaping time," you also increase the number of impulses used and you add constraints to force the sensitivity to be small.
It seems clear that you want the SD shaper to be insensitive to parameter mismatches, but you do not make that a stated goal. This goal needs to be stated clearly at the very beginning of the paper.

Answer: According to the reviewer’s comment, I clarified the goal of the paper by adding the following sentence at the Abstract and at the end of the Introduction section.

“As the specified shaping time increases, the required number of impulses of the SD shaper increases one by one according to the number of added derivative constraints for better robustness to modeling errors.”

Additionally, some sentences were modified to clarify the goal of the paper.

This additional, but almost unmentioned, constraint leads to other problems. For example, if one wants the duration of an input shaper to be some number of half-periods of the system’s response, you can design an input shaper with two impulses. This is not mentioned -- and, in fact, in equation (6), you seem to say otherwise. In line 136, you state that you do not design input shaper with N=3 when Ts > 1 even though you could because they are quite sensitive to modeling error, but this was never an "official" constraint.

Answer: According to the reviewer’s comment, I added the following phrase and sentence at the end of the paragraph after Eq. (6).

“However, this input shaper is not a satisfactory solution because of its high sensitivity to modeling errors compared to SD shapers.”

There exists only one input shaper, ZV shaper, with two impulses. This fact is obvious in view of impulse vector diagram. For the input shaper with a shaping time less than half period, we need a negative impulse and thus we do not consider it in this paper. This is explained at the last paragraph below Eq. (26), which is “Moreover, if the dimensionless shaping time is less than 0.5, we require negative impulses to remove residual vibrations. Thus, SD shapers with $T_{s}<0.5$ were not considered in this study.”

My second major concern has to do with the “design rules” for the SD shaper. If I understood what you did correctly, you designed a series of input shapers by specifying a duration and then choosing a number of impulses and relevant constraints that experience showed worked well. You made sure that you had one “free” coefficient, and iterated on it to find the value that led your SD shaper to be as insensitive to parameter mismatch as possible. If this is correct, you should describe the technique first, and you should explain how you chose the number of impulses – since fewer impulses could have been used.
Also, as things stand, the design rules seem very “ad hoc.” If you can provide a better explanation for where they come from and how you decided how many impulses to use, that would be most helpful to the reader and would strengthen the paper.

Answer: According to the reviewer’s comment, I added the following paragraph at the end of the paragraph after Eq. (6).

“Then the design procedure of SD shapers can be summarized as follows:

(i) Determine the number of impulses *N* from the specified shaping time using Eq (6).

(ii) Obtain constraint equations for the SD shaper.

(iii) Find impulse magnitudes and times $A_{i},t_{i}$ by solving constraint equations analytically or numerically, in which the last impulse magnitude $A_{N}$ is a free parameter to maximize the robustness to modeling errors.”

Also, I added one more sentence after Fig. 2 as follows.

“$\theta_{3}$ is given from the specified shaping time and $I_{3}$ is given from the free parameter $A_{3}$ that is used for maximizing the robustness to modeling errors.”

The reason for the number of impulses is explained in the paragraph below Eq. (6). We know that ZV shaper has $T_{s}=0.5$, ZVD shaper has $T_{s}=1$, ZVDD shaper has $T_{s}=1.5$, and ZVDDD shaper has $T_{s}=2$. By the way, if $0.5<T_{s}\leq1$, we can draw an impulse vector diagram for the SD shaper as shown in Fig. 2, therefore this SD shaper is composed of three impulses, which means we have five parameters $A_{1},A_{2},A_{3},t_{2},t_{3}$. Among five parameters, $t_{3}$ is given from the specified shaping time, and thus we have four unknowns $A_{1},A_{2},A_{3},t_{2}$. If we choose $A_{3}$ as a free parameter for minimizing sensitivity to modeling errors (i.e., we select arbitrary value $A_{3}$ between 0 to 1), we only have three unknowns $A_{1},A_{2},t_{2}$. Thus, we need three equations to find these three unknowns. Those are two residual vibration constraints and one normalization constraint. Similarly, if $1<T_{s}\leq1.5$, we can draw an impulse vector diagram for the SD shaper as shown in Fig. 3, and this SD shaper is composed of four impulses. Then we have five unknowns $A_{1},A_{2},A_{3},t_{2},t_{3}$ because $A_{4}$ is given by the designer. These five unknowns can be obtained by adding two more derivative constraints.

To include the above explanation for the selection of the number of impulses, I revised the paragraph below Eq. (6) as follows.

“Eq (6) can be explained as follows. If $T_{s}$ is between 0.5 and 1, the angle of the final impulse vector is in $\pi<\theta_{N}\leq2\pi$, and thus an impulse vector diagram with zero resultant can be drawn using three impulse vectors (minimum number of impulse vectors) as shown in Fig 2. Therefore, three impulses are required. Three impulses have four unknowns $A_{1},A_{2},A_{3},t_{2}$ since $t_{3}$ is given from the specified shaping time. These four unknowns can be obtained by solving two residual vibration constraints and one normalization constraint with $A_{3}$ chosen in advance by the designer. If $T_{s}$ is between 1 and 1.5, the angle of the final impulse vector is in $2\pi<\theta_{N}\leq3\pi$, and an impulse vector diagram with zero resultant can be drawn using four impulse vectors as shown in Fig 3. Therefore, four impulses are required. Four impulses can be obtained by solving two residual vibration constraints, two derivative constraints, and a normalization constraint simultaneously (six unknowns $A_{1},A_{2},A_{3},A_{4},t_{2},t_{3}$ with $A_{4}$ chosen in advance by the designer). Similarly, if $T_{s}$ is between 1.5 and 2, five impulses are required, and these five impulse can be obtained by solving two residual vibration constraints, two derivative constraints, two second-order derivative constraints, and a normalization constraint (eight unknowns $A_{1},A_{2},A_{3},A_{4},A_{5},t_{2},t_{3},t_{4}$ with $A_{5}$ chosen in advance by the designer) [13,14].

Using the final impulse magnitude $A_{N}$ as a free parameter to minimize the sensitivity to modeling errors, we can maximize the robustness to modeling errors (e.g., 5% insensitivity) in the design of SD shapers. Note that considering the impulse vector diagram in case of $T_{s}>1$, an input shaper with three impulses can be also designed because the sum of the impulse vectors can be made zero. However, this input shaper is not a satisfactory solution because of its high sensitivity to modeling errors compared to SD shapers.”

I would like to raise several somewhat more minor issues as well.

On line 32, you speak of two researchers as being at MIT. As far as I can tell, neither is at MIT today.

Answer: According to the reviewer’s comment, I omitted “at MIT”.

On line 54, I think that “conventionally” should be replaced with a more suitable word.

Answer: According to the reviewer’s comment, I revised it as follows,

“Conventionally -> In the 1990s”

Equations (1) and (2) did not print properly in the PDF.

Answer: It seems it is a software problem of pdf printer. I corrected it as best as I can.

You seem to assume a second order system. As I think that this is actually part of what sets the number of impulses, this assumption should also be made clear quite early on. If you have also examined systems with more than one pair of complex poles, you can say a bit about such systems too.

Answer: According to the reviewer’s comment, I added the following sentence at the beginning of ‘Design of SD shaper’ section.

“Vibratory system dynamics is assumed to be an underdamped second-order system $\omega_{n}^{2}/(s^{2}+2\zeta\omega_{n}s+\omega_{n}^{2})$ where $\omega_{n}$ is the undamped natural frequency and $\zeta$ is the damping ratio of the system.”

On lines 93-96 you give a description of the Dirac delta function. I think this description can be omitted.

Answer: According to the reviewer’s comment, I omitted the following paragraph.

“The function value of the impulse function is infinite at time $t_{i}$ and 0 at other times, and integral value becomes 1 when integrated from $- \infty$ to $\infty$. An impulse function with impulse magnitude $A_{i}$ and impulse time $t_{i}$ can be expressed as Dirac delta function $A_{i} \delta(t-t_{i})$.”

Is it well known that your non-linear design equations always have a solution? If it is, it would be nice to state the fact and give a reference to it.

Answer: According to the reviewer’s comment, I clarified the existence of the solution satisfying all the nonlinear constraint at the last sentence of the last paragraph below Eq. (26) as follows.

“Note that the SD shaper satisfying all nonlinear constraints always exists because an impulse vector diagram satisfying all the nonlinear constraints of the SD shaper can be always drawn geometrically and then the SD shaper can be obtained from the impulse vector diagram.”

[Kang CG. Impulse vectors for input-shaping control: A mathematical tool to design and analyze input shapers. IEEE Control Systems Magazine. 2019;39(4):40-55]

On line 129 and in other places, you speak of having a “predetermined” value. It seems that what you mean is that this value can be chosen by the designer, and when you later step through values of a parameter, it is this parameter that you change. If this is correct, then I think that the word “predetermined” is somewhat misleading and should be changed.

Answer: According to the reviewer’s comment, ‘predetermined’ is changed to ‘chosen in advance by the designer’

Your conclusion also does not mention insensitivity to changes in the system’s parameters. As dealing with this issue is one of the design constraints, it should be mentioned in the conclusion as well.

Answer: According to the reviewer’s comment, I added the following sentence at the end of the first paragraph of the Conclusion.

“As the specified shaping time increases, the required number of impulses of the SD shaper increases one by one according to the number of added derivative constraints for better robustness to modeling errors.”

Overall, I enjoyed the article, but its goals must be stated precisely and the design rules and their motivation must be explained clearly. I also think that the article could also be shortened somewhat. There are more examples of how to calculate SD shapers than seem necessary to me.

Answer: Thank you for your valuable comments.

**Response to Reviewer #2**

Reviewer #2: This paper is one application of impulse vectors for input-shaping control. The finding is predictable, but may be useful for some applications that require limited response time, and of course, the vibration suppression performance is limited. In comparison to traditional IS techniques such as ZV, ZVD, more computation is required. But it helps readers to understand the input shaping technique more clearly. It also shows the effectiveness of impulse vector approach to generalize the development of input shaping technique.

Answer: Thank you for valuable response.

**Response to Reviewer #3**

Reviewer #3: The paper aims to define a particular class of Input shaping filters with a specified-duration (SD). Instead, classical input shapers only take into account the characteristics of the system to which they are applied. So the total duration of these shapers is not a free parameter for the designer. First, the procedure to define the SD shapers is defined, then these filters are analyzed with both a numerical and an experimental approach. The main contributions of this work consist in the definition of the SD shapers.

I will focus only on the contents of the paper and not on its form. The article is interesting, but I think that some improvements are necessary before publication.

1) Lines 39/40: “The vibratory system in Fig. 1 includes a feedback loop and a feedback controller”. I think it could be useful to show the feedback loop also in the figure, since talking about it without showing it can confuse the reader.

Answer: According to the reviewer’s comment, I redrew Fig. 1 and replaced it as follows.

2) Lines 55/57: “The SI shaper was designed to ensure that the sensitivity function V of the input shaper was within 0.05 for a specified range of modelling errors”. The value 0.05 (i.e. 5%) is only exemplary. An SI shaper can be modeled with other percentages of admitted sensitivity. I think it is better to use in all the text (not only in these lines) a generic value (e.g. called z) instead of 0.05 and to specify the entity of this value only when it is strictly necessary: e.g. in the numerical examples to calculate the parameters of the shapers it is necessary to use z = 0.05 (or z equal to another numeric value).

Answer: According to the reviewer’s comment, I revised lines 55/65 as follows.

“The SI shaper was designed to ensure that the sensitivity function V of the input shaper was within a tolerance value $V_{tol}$ for a specified range of modeling errors. The sensitivity function V indicates the relative magnitude of residual vibration for modeling errors in natural frequency and damping ratio. Thus, to design an SI shaper with, for example, $V_{tol}=0.05$, we first specify an arbitrary 5% insensitivity $I_{0.05}$, which forms the range of modeling errors such that the relative residual vibration is within 5%, and then design the input shaper to satisfy this $I_{0.05}$ condition. The 5% insensitivity $I_{0.05}$ represents the insensitivity (or robustness) to modeling errors of the input shaper when $V_{tol}=0.05$.

The shaping time (or shaper duration) as well as the robustness to modeling errors are important factors in the design of input shapers.”

3) Lines 67/69: “Although the shaping time is an important factor in the design of input shapers, no studies have been conducted on designing input shapers that can satisfy arbitrarily specified shaping times”. In [1,2] classical input shapers (ZV, ZVD, …) are used to obtain motion laws with a prescribed duration. The procedure adopted in this work is very different since here the shaper is directly designed with a prescribed duration. In [1,2], instead, the shapers are computed, and then, by knowing their duration, the motion law in input to the shaper is found in such a way to execute the movement in the right total time. Even if the method is different, I think that the final aim of the input shapers defined in this manuscript could be the same obtained in [1,2]. With this in mind, I think an analysis of the differences between the method adopted in this work and the technique applied in [1,2] could be interesting. Is the solution proposed in this paper better/worse/indifferent?

[1] Boscariol, P., and Richiedei, D., 2018, “Robust Point-to-Point Trajectory Planning for Non-Linear Underactuated Systems: Theory and Experimental Assessment,” Rob. Comput. Integr. Manuf., 50, pp. 256–265.
[2] Guagliumi, L., Berti, A., Monti, E., and Carricato, M.: Anti-Sloshing Trajectories for HighAcceleration Motions in Automatic Machines. ASME, Journal of Dynamic Systems, Measurement, and Control (2022). doi: 10.1115/1.4054224.

Answer: Thanks for the information on the research works by Boscariol et al.[1] and Guagliumi et al.[2]. I’ve read these two papers interestingly, and I cited these two papers in my manuscript. However, the comparison research between these papers’ logic and my manuscript’s logic will take time and need efforts, so I will leave it as a further research topic.

4) Line 92. I suggest writing relation ω_d=ω_n √(1-ζ^2 ) as an equation and not writing it in the text.

Answer: According to the reviewer’s comment, I revised $\omega_{d}$ as an equation in a separate line as follows.

“and $\omega_{d}$ is the damped natural frequency of the system, which is defined by

$\omega_{d} =\omega_{n}\sqrt{1-\zeta^{2}}$.”

5) Lines 120/134: “If the dimensionless shaping time Ts is between 0.5 and 1 (that is, between half and a period), the SD shaper consists of three impulses. If Ts is between 1 and 1.5, the SD shaper consists of four impulses, and if it is between 1.5 and 2, it consists of five impulses. …” Why? It is not clear how the number of impulses is chosen, but this is an important point in the definition of the SD shaper. It is necessary to better explain the reason behind the choice of the number of impulses.

Answer: The reason for the number of impulses is explained in the paragraph below Eq. (6). We know that ZV shaper has $T_{s}=0.5$, ZVD shaper has $T_{s}=1$, ZVDD shaper has $T_{s}=1.5$, and ZVDDD shaper has $T_{s}=2$. By the way, if $0.5<T_{s}\leq1$, we can draw an impulse vector diagram for the SD shaper as shown in Fig. 2, therefore this SD shaper is composed of three impulses, which means we have five parameters $A_{1},A_{2},A_{3},t_{2},t_{3}$. Among five parameters, $t_{3}$ is given from the specified shaping time, and thus we have four unknowns $A_{1},A_{2},A_{3},t_{2}$. If we choose $A_{3}$ as a free parameter for minimizing sensitivity to modeling errors (i.e., we select arbitrary value $A_{3}$ between 0 to 1), we only have three unknowns $A_{1},A_{2},t_{2}$. Thus, we need three equations to find these three unknowns. Those are two residual vibration constraints and one normalization constraint. Similarly, if $1<T_{s}\leq1.5$, we can draw an impulse vector diagram for the SD shaper as shown in Fig. 3, and this SD shaper is composed of four impulses. Then we have five unknowns $A_{1},A_{2},A_{3},t_{2},t_{3}$ because $A_{4}$ is given by the designer. These five unknowns can be obtained by adding two more derivative constraints.

According to the reviewer’s comment, I revised the explanation below Eq. (6) as follows.

“Eq (6) can be explained as follows. If $T_{s}$ is between 0.5 and 1, the angle of the final impulse vector is in $\pi<\theta_{N}\leq2\pi$, and thus an impulse vector diagram with zero resultant can be drawn using three impulse vectors (minimum number of impulse vectors) as shown in Fig 2. Therefore, three impulses are required. Three impulses have four unknowns $A_{1},A_{2},A_{3},t_{2}$ since $t_{3}$ is given from the specified shaping time. These four unknowns can be obtained by solving two residual vibration constraints and one normalization constraint with $A_{3}$ chosen in advance by the designer. If $T_{s}$ is between 1 and 1.5, the angle of the final impulse vector is in $2\pi<\theta_{N}\leq3\pi$, and an impulse vector diagram with zero resultant can be drawn using four impulse vectors as shown in Fig 3. Therefore, four impulses are required. Four impulses can be obtained by solving two residual vibration constraints, two derivative constraints, and a normalization constraint simultaneously (six unknowns $A_{1},A_{2},A_{3},A_{4},t_{2},t_{3}$ with $A_{4}$ chosen in advance by the designer). Similarly, if $T_{s}$ is between 1.5 and 2, five impulses are required, and these five impulse can be obtained by solving two residual vibration constraints, two derivative constraints, two second-order derivative constraints, and a normalization constraint (eight unknowns $A_{1},A_{2},A_{3},A_{4},A_{5},t_{2},t_{3},t_{4}$ with $A_{5}$ chosen in advance by the designer) [13,14].

Using the final impulse magnitude $A_{N}$ as a free parameter to minimize the sensitivity to modeling errors, we can maximize the robustness to modeling errors (e.g., 5% insensitivity) in the design of SD shapers. Note that considering the impulse vector diagram in case of $T_{s}>1$, an input shaper with three impulses can be also designed because the sum of the impulse vectors can be made zero. However, this input shaper is not a satisfactory solution because of its high sensitivity to modeling errors compared to SD shapers.”

6) Lines 158/159: “Among them, A1, A2, A3, t2 with the biggest 5% insensitivity I0.05 is selected as the SD shaper.” I think this sentence confuses the reader since the explanation of how these values are chosen to maximize the insensitivity of the shaper is given in the following. I suggest omitting this sentence.

Answer: According to the reviewer’s comment, I omitted that sentence and revised it as follows.

“For a given $\theta_{3}$, we assume a specific value of the final impulse magnitude $A_{3}$ $(0<A_{3}<1)$, and find $A_{1}, A_{2}, t_{2}$ that satisfy Eqs (7)–(9). Then change the value of $A_{3}$, and find $A_{1}, A_{2}, t_{2}$ that satisfy Eqs (7)–(9), repeatedly.”

7) Lines 197/203. Is it possible to find an analytical solution to the problem of finding the shaper that maximizes the insensitivity? Maybe by adding another constraint to the original ones in equations (7)-(9) to find the value of A3. The resulting system could be solved also numerically. If this is not possible, this should be discussed: why is it not possible?

Answer: It is not possible to find an analytical solution that maximizes the 5% insensitivity because the 5% insensitivity $I_{0.05}$ cannot be obtained analytically ($I_{0.05}$ can be obtained only by numerical computations).

8) Line 252. In equation (23), constraints 3 and 4 should be clarified. It is mentioned that it is necessary to add derivative constraints, but more specifically, how these constraints are computed? I think they are the derivative of constraints 1 and 2 with respect to ω_n, but this should be clarified. Idem in Eq (25).

Answer: In Eq (23), constraints 3 and 4 are obtained as follows.

Let

$$\begin{matrix} C(\omega_{n})=\sum_{i=1}^{N} A_{i}e^{\zeta\omega_{n}t_{i}}\cos(\omega_{d}t_{i}) \cdots\cdots\cdots(1) \\ S(\omega_{n})=\sum_{i=1}^{N} A_{i}e^{\zeta\omega_{n}t_{i}}\sin(\omega_{d}t_{i}) \cdots\cdots\cdots(2) \end{matrix}$$

Then

$\frac{dC}{d\omega_{n}}=\sum_{i=1}^{N} \left[A_{i} \left(\zeta t_{i}\right) e^{\zeta\omega_{n} t_{i}}\cos\omega_{d}t_{i}-A_{i} e^{\zeta\omega_{n} t_{i}}\left(t_{i}\sqrt{1-\zeta^{2}}\right)\sin\omega_{d}t_{i}\right]=0$ …… (3)

$\frac{dS}{d\omega_{n}}=\sum_{i=1}^{N} \left[A_{i} \left(\zeta t_{i}\right) e^{\zeta\omega_{n} t_{i}}\sin\omega_{d}t_{i}+A_{i} e^{\zeta\omega_{n} t_{i}}\left(t_{i}\sqrt{1-\zeta^{2}}\right)\cos\omega_{d}t_{i}\right]=0$ …….. (4)

Now the following computation will give us the constraint 3 and 4 of Eq (23).

$$\begin{matrix} (3)\times\zeta+ (4)\times\sqrt{1-\zeta^{2}} \to\text{Contraint 3 of Eq (23)} \\ (4)\times\zeta- (3)\times\sqrt{1-\zeta^{2}} \to\text{Contraint 4 of Eq (23)} \end{matrix}$$

Similarly, we can obtain second-order derivative constraint in Eq (25).

So, according to the reviewer’s comment, I added the following explanations below Eq. (23) and (25).

“Two derivative constraints, the third and fourth of Eq (23), are obtained by differentiating two residual vibration constraints, the first and second of Eq (23), with respect to $\omega_{n}$. The first and second constraints of Eq (23) can be expressed in the form of functions of $\omega_{n}$ as follows.

$$\begin{matrix} C(\omega_{n})=A_{1}+A_{2}e^{\zeta\omega_{n}t_{2}}\cos(\omega_{d}t_{2})+A_{3}e^{\zeta\omega_{n}t_{3}}\cos(\omega_{d}t_{3})+A_{4}e^{\zeta\omega_{n}t_{4}}\cos(\omega_{d}t_{4})=0 \\ S(\omega_{n})=A_{2}e^{\zeta\omega_{n}t_{2}}\sin(\omega_{d}t_{2})+A_{3}e^{\zeta\omega_{n}t_{3}}\sin(\omega_{d}t_{3})+A_{4}e^{\zeta\omega_{n}t_{4}}\sin(\omega_{d}t_{4})=0 \end{matrix}$$

Then we can obtain two equations, $dC / d\omega_{n}=0$ and $dS / d\omega_{n}=0$ by differentiating $C (\omega_{n})$ and $S (\omega_{n})$ with respect to $\omega_{n}$. These two equations are equivalent to the third and fourth constraints of Eq (23).”

Also, I added the following explanation below Eq. (25).

“Two second-order derivative constraints, the fifth and sixth of Eq (25), is obtained by differentiating two derivative constraints, the third and fourth of Eq (25), with respect to $\omega_{n}$.”

9) Lines 299-300: “Moreover, if the dimensionless shaping time is less than 0.5, we require negative impulses to remove residual vibrations.” Why? It would be interesting to explain the reason behind this consideration.

Answer: Because the angle of the final impulse vector is less than $\pi$, and thus the second impulse vector among three must be negative to make the resultant to be zero as shown in the figure below.

Negative impulse vector $\mathbf{I}_{2}$ corresponds to negative impulse of the input shaper. For the impulse vector, refer to the paper, [Kang CG. Impulse vectors for input-shaping control: A mathematical tool to design and analyze input shapers. IEEE Control Systems Magazine. 2019;39(4):40-55]

10) Lines 324/325: “If no modeling error exists, then the SD shaper has the shaping time of exactly 0.3 s as shown in Fig 5A”. I thought the duration of the shaper was a free parameter chosen by the designer. If there are errors in the modeling of ω_n, this should not interfere with the duration of the shaper. Am I right? If yes, I would omit this sentence that confuses the reader. Idem for lines 354/355.

Answer: I wanted to describe in that sentence that the simulation result shows exactly 0.3 s shaping time as designed in Eq (21). I think description of the simulation results for the cases with and without modeling errors is helpful for the reader who is not familiar with this topic. If I omit that sentence, only the simulation result for the case with modeling error is described. So I revised that sentence as follows.

“If no modeling error exists, then the SD shaper has the shaping time of exactly 0.3 s as designed in Eq (21).”

Also, I revised that sentence in lines 354/355 as follows.

“If no modeling error exists, the SD shapers exhibit the exact shaping time of 0.3, 0.6 and 0.85 s, respectively, as designed in Eqs (22), (24) and (26).”

11) Line 409/410: “The obtained three SD shapers were simulated using Simulink, and the results are shown in Fig 10”. I think it would be interesting to show also in the experimental results a comparison between the SD shaper and the classical ZV/ZVD as previously done for the numerical results related to the sensitivity analysis.

Answer: I borrowed the experimental apparatus from Prof. Ha and Prof. Kang for testing the SD shaper performance experimentally. Please excuse me that I cannot borrow it again for comparison works between the SD shaper and ZV/ZVD shapers. Instead, I found two papers that show the experimental results of ZV and ZVD shaper using the same experimental apparatus.

For ZV shaper: C.-G. Kang and R. Hassan, “Design and analysis of input shapers using impulse vectors,” *Journal of Institute of Control, Robotics and Systems*, 26(1):1-8, 2020. (in Korean)

For ZVD shaper: C.-G. Kang and M.-T. Ha, “On a sensitivity function of input shaping control,” *Proc. of the 9^th^ International Conference on Ubiquitous Robots and Ambient Intelligence*, pp. 380-384, Daejeon, Korea, Nov. 26-29, 2012.

Some improvements are necessary before the publication. In particular, I think it is very important to be more clear on point 5) in my list since the choice of the number of impulses of the input shaper is one of the most important factors in the definition of the shaper, but in my opinion here this aspect is not clear.

Answer: According to the reviewer’s comment, I revised the manuscript for improvement, especially for clarifying the choice of the number of impulses of the SD shapers using long paragraph. Thank you for your valuable comments.

**Response to Reviewer #4**

Reviewer #4: The paper suggest a new input shaper with arbitrarily specified shaping times, a specified-duration shaper. The author test their proposed shaper using numerical simulation of a second-order system and validate it experimentally by suppressing the induced residual vibration of a horizontal beam. The paper is not recommended to be published in this form for mainly two reasons; (1) the novelty of the proposed shaper where several shaped commands using optimal control and command shaping were recently developed with robustness adjustability, smoothness enhancement, and variable command time length, and (2) the presentation of the paper including its writing style and structure. For instance, the author can consult the following papers for more details:

“Robust time-delay control of multimode systems” by Singh and Vadali.
“Discrete-time command profile for simultaneous travel and hoist maneuvers of overhead cranes” by Alghanim et. al.

Here are my comments to the authors:

The readers of published papers are aware of the vibration types so no need to mention that there free and force vibration in page 2 line 24.

Answer: According to the reviewer’s comment, I omitted “(either forced vibrations or free vibrations)” in page 2 line 24.

There is no need to mention that Smith was at UC Barkeley when he introduced the posicast control; page 2 line 32.
The same previous comment goes with Singer and Singhose at MIT.

Answer: According to the reviewer’s comment, I removed “at UC Berkeley” and “at MIT” in page 2 line 32, and revised as follows.

“Input-shaping control was first introduced as posicast control in the 1950s by O. J. M. Smith [11]. Since 1980s, N. Singer, W. Singhose, and T. Singh have considerably improved input-shaping control by adding derivative constraints [12-15].’

Page 2 line 33; I am not sure the readers will know what the derivative constraints is. The author could simply state the Singer and Singhose enhance the robustness.

Answer: According to the reviewer’s comment, I added some more explanations about derivative constraints below Eq. (23) as follows.

“Two derivative constraints, the third and fourth of Eq (23), are obtained by differentiating two residual vibration constraints, the first and second of Eq (23), with respect to $\omega_{n}$. The first and second constraints of Eq (23) can be expressed in the form of functions of $\omega_{n}$ as follows.

$$\begin{matrix} C(\omega_{n})=A_{1}+A_{2}e^{\zeta\omega_{n}t_{2}}\cos(\omega_{d}t_{2})+A_{3}e^{\zeta\omega_{n}t_{3}}\cos(\omega_{d}t_{3})+A_{4}e^{\zeta\omega_{n}t_{4}}\cos(\omega_{d}t_{4})=0 \\ S(\omega_{n})=A_{2}e^{\zeta\omega_{n}t_{2}}\sin(\omega_{d}t_{2})+A_{3}e^{\zeta\omega_{n}t_{3}}\sin(\omega_{d}t_{3})+A_{4}e^{\zeta\omega_{n}t_{4}}\sin(\omega_{d}t_{4})=0 \end{matrix}$$

Then we can obtain two equations, $dC / d\omega_{n}=0$ and $dS / d\omega_{n}=0$ by differentiating $C (\omega_{n})$ and $S (\omega_{n})$ with respect to $\omega_{n}$. These two equations are equivalent to the third and fourth constraints of Eq (23).”

Also, I added the following explanation below Eq. (25).

“Two second-order derivative constraints, the fifth and sixth of Eq (25), is obtained by differentiating two derivative constraints, the third and fourth of Eq (25), with respect to $\omega_{n}$.”

Omit the sentence “in natural frequency and damping ratio” in page 2 line 37.

Answer: According to the reviewer’s comment, I omitted that sentence.

The last sentence in the second paragraph is redundant.
The open-loop control is also sensitive to external disturbance.

Answer: According to the reviewer’s comment, I omitted that sentence.

The last sentence in the third paragraph is redundant.

Answer: I will leave it for the reader who is not familiar with input shaping control.

The first and second sentences is page 3 (lines 47 and 48) are redundant.

Answer: Those are a part of the caption of Fig. 1 (not text). I followed the figure & caption format instructed in the homepage guideline.

The paragraph starting from the line 55 in page is confusing since the readers may not know what the sensitivity function V is.

Answer: The sensitivity function is explained in Eqs. (19) and (20).

The introduction is quite weak and need to be improved. The author should survey through recent papers in command and input shapings to further demonstrate the novelty of the present results.

Answer: According to the reviewer’s comment, the Introduction is improved by citing more recent research works, and by adding and revising some sentences.

The equations (1) and (2) are only limited to underdamped system.

Answer: According to the reviewer’s comment, I assumed an underdamped system at the beginning of the section, Design of an SD shaper, as follows.

“In this section, the design of an SD shaper with an arbitrarily specified shaping time $t_{s}$ is described. Vibratory system dynamics is assumed to be an underdamped second-order system $\omega_{n}^{2}/(s^{2}+2\zeta\omega_{n}s+\omega_{n}^{2})$ where $\omega_{n}$ is the undamped natural frequency and $\zeta$ is the damping ratio of the system. An impulse vector $\mathbf{I}_{i} (i=1,2,\cdots,N)$ with magnitude $I_{i}$ and angle $\theta_{i}$ in the polar coordinate plane was proposed by Kang [13], which is as follows:”

The sentences starting from line 93 should be omitted because it is quite obvious what impulses are and what dirac delta function is.

Answer: According to the reviewer’s comment, I omitted the following paragraph.

“The function value of the impulse function is infinite at time $t_{i}$ and 0 at other times, and integral value becomes 1 when integrated from $- \infty$ to $\infty$. An impulse function with impulse magnitude $A_{i}$ and impulse time $t_{i}$ can be expressed as Dirac delta function $A_{i} \delta(t-t_{i})$.”

The equations (4) and (5) are obvious and should be omitted.

Answer: I will leave them for clarity for the reader who is not familiar with this topic.

The paragraph starting from line 127 should be rephrased.

According to the reviewer’s comment, I rephrased the paragraph below Eq. (6) as follows.

“Eq (6) can be explained as follows. If $T_{s}$ is between 0.5 and 1, the angle of the final impulse vector is in $\pi<\theta_{N}\leq2\pi$, and thus an impulse vector diagram with zero resultant can be drawn using three impulse vectors (minimum number of impulse vectors) as shown in Fig 2. Therefore, three impulses are required. Three impulses have four unknowns $A_{1},A_{2},A_{3},t_{2}$ since $t_{3}$ is given from the specified shaping time. These four unknowns can be obtained by solving two residual vibration constraints and one normalization constraint with $A_{3}$ chosen in advance by the designer. If $T_{s}$ is between 1 and 1.5, the angle of the final impulse vector is in $2\pi<\theta_{N}\leq3\pi$, and an impulse vector diagram with zero resultant can be drawn using four impulse vectors as shown in Fig 3. Therefore, four impulses are required. Four impulses can be obtained by solving two residual vibration constraints, two derivative constraints, and a normalization constraint simultaneously (six unknowns $A_{1},A_{2},A_{3},A_{4},t_{2},t_{3}$ with $A_{4}$ chosen in advance by the designer). Similarly, if $T_{s}$ is between 1.5 and 2, five impulses are required, and these five impulse can be obtained by solving two residual vibration constraints, two derivative constraints, two second-order derivative constraints, and a normalization constraint (eight unknowns $A_{1},A_{2},A_{3},A_{4},A_{5},t_{2},t_{3},t_{4}$ with $A_{5}$ chosen in advance by the designer) [13,14].

Using the final impulse magnitude $A_{N}$ as a free parameter to minimize the sensitivity to modeling errors, we can maximize the robustness to modeling errors (e.g., 5% insensitivity) in the design of SD shapers. Note that considering the impulse vector diagram in case of $T_{s}>1$, an input shaper with three impulses can be also designed because the sum of the impulse vectors can be made zero. However, this input shaper is not a satisfactory solution because of its high sensitivity to modeling errors compared to SD shapers.”

No need to mention what kind of software the author used since the iterative problem is quite easy and straightforward.
The something goes when the author stated that they used the MATLAB built-in function fsolve to solve the nonlinear problems. I am quite sure that the resultant system of nonlinear equations are not really complex and so need to explicitly state what kind of solver the author used to solve for the roots.

Answer: Partially I agree to the reviewer’s comment, but I want to leave the explanation about fsolve() function because it helps the reader to regenerate it quickly.

The SP shapers were only designed when 0.5<t_s Write the command duration of the ZV and ZVD in all the figures to have reasonable comparison.

Answer: Obviously, the shaping time of ZV and ZVD shaper is longer than the SD shaper with three impulses and with four impulses, respectively. I borrowed the experimental apparatus from Prof. Ha and Prof. Kang for testing the SD shaper performance experimentally. Please excuse me that I cannot borrow it again for comparison works between the SD shaper and ZV/ZVD shapers. Instead, I found two papers that show the experimental results of ZV and ZVD shaper using the same experimental apparatus.

For ZV shaper: [C.-G. Kang and R. Hassan, “Design and analysis of input shapers using impulse vectors,” *Journal of Institute of Control, Robotics and Systems*, 26(1):1-8, 2020. (in Korean)]

For ZVD shaper: [C.-G. Kang and M.-T. Ha, “On a sensitivity function of input shaping control,” *Proc. of the 9^th^ International Conference on Ubiquitous Robots and Ambient Intelligence*, pp. 380-384, Daejeon, Korea, Nov. 26-29, 2012.]

Provide a thorough numerical experiment and summarize the findings at the end of the numerical and experiment sections.

Answer: According to the reviewer’s comment, I added a sentence for the findings by simulation and experimental studies in the below Table 1 (Experimental demonstrations section).

“As the specified shaping time is increased, the actual shaping time is also increased in both simulation and experimental results as expected. In Fig 10, the experimental …”

Moreover, I modified some sentences in the simulation analysis section.

The paper can be further improved if the author attach the general case where special cases can be derived from the general case. The author should also read and survey the recent contributions of controlling techniques especially the ones dealing with the input and command shaping. The language and the presentation of the paper should improved. Obvious phrases, simple equations, and repeated sentences should be omitted in order to make the reader interesting when he/she is reading the paper. To make it simple, the proposed shaper could be published but the author should try to convince the reviewers the novelty of his shaper and the contribution of his findings.</t_s

Answer: According to the reviewer’s comment, I added two more references related to this draft’s work, and improved English and presentation as best as I can. Thank you for your valuable comments.

[English language editing for the manuscript was done through Editage, a professional editing service (certificate number: CDNBY_1_2).]
