# Supplementary material for: Specified-duration shapers for suppressing residual vibrations
Source: PLoS One. 2022 Nov 28;17(11):e0276669. doi: 10.1371/journal.pone.0276669 (PMC9704665; doi:10.1371/journal.pone.0276669)
Supplement: Attachment — Submitted filename: Response to Reviewers_2.docx [file pone.0276669.s002.docx]

**Response to Reviewer #1**

**Reviewer #1:** 
I read your paper with interest. I still feel that the point of the paper is not being presented well. You are presenting a method to design a controller that is both specified duration and insensitive to changes, yet you call your controller an SD controller and do not stress that it is also required to be relatively insensitive. Because you do not stress that the system must be insensitive, many of the choices you make are hard to understand.

One place you could stress the “robustness” issue is on line 15. There you refer to the shaping time. I think it would be best to refer to the shaping time and to the robustness.

Answer: According to the reviewer’s comment, I revised the line 16 as follows.

In this study, a specified-duration (SD) shaper, which is an input shaper with maximal robustness to modeling errors for an arbitrarily specified shaping time, and …

I would strongly recommend changing the name of the controller. SD puts all the emphasis on the duration and none on the “robustness.” I think that if you change the emphasis to include robustness, you will help the reader understand what you are doing.

Answer: Thanks for your comment, but I think the most important contribution of this paper is to design an input shaper with an *arbitrarily specified* shaping time (shaper duration), so I’d like to keep this name, *specified-duration* (SD) shaper with your understanding. However, according to your valuable comment, I revised several sentences to emphasize the robustness issue in the following sections.

Abstract (line 16): In this study, a specified-duration (SD) shaper, which is an input shaper with maximal robustness to modeling errors for an arbitrarily specified shaping time, and …

Introduction (line 75): In this paper, a novel specified-duration (SD) shaper that satisfies an arbitrarily specified shaping time with maximal robustness to modeling errors is proposed.

Design of an SD shaper (line 90): In this section, the design of an SD shaper with maximal robustness to modeling errors for an arbitrarily specified shaping time $t_{s}$ is described.

Conclusion (line 506): This paper introduced an SD shaper that can suppress residual vibrations at an arbitrarily specified shaping time with maximal robustness to modeling errors.

From roughly line 140 to line 155, you describe some of your design rules. On lines 141 and 144 you state that four or five (respectively) impulses are, _therefore_ [my emphasis], required. On lines 150 – 153, you state that you can actually manage with three impulses. Thus, four or five impulses are not required, and the word “therefore” is misleading.

Answer: According to your comment, I rephrased the sentence in line 143 after removing “therefore” sentence as follows.

…and an impulse vector diagram with zero resultant can be drawn using four impulse vectors as shown in Fig 3, which corresponds to an input shaper with four impulses. ~~Therefore, four impulses are required.~~

On lines 152 and 153, you state that the three impulse solution is unacceptable because it is not sufficiently insensitive. Once again, we see that it is critical that you state that low-sensitivity is an absolute requirement from your input shaper. Then you must explain or demonstrate that the three impulse solution is too sensitive to modeling errors.
If I understand what you are trying to do correctly, you want an insensitive specified duration shaper. It seems that experience/experiments have shown how many impulses are necessary in order to have enough degrees of freedom to achieve all your goals. If this is the case, please say so and provide theoretical or experimental support your statement. Currently, it is unclear how you arrived at (6).

Answer: According to the reviewer’s comment, I added the following explanation including one new figure and table at the end of the section ‘Analysis of SD shapers’ as follows.

If $t_{s}=0.85\text{ s}$ for the underdamped system with $\hat{\omega}_{n}=2\mathbf{ }\text{Hz}$ and $\zeta=0.1$, the dimensionless shaping time is $T_{s}\approx1.692>1.5$, and thus the SD shaper is composed of five impulses according to (7). However, considering the impulse vector diagram for the case with $T_{s}>1.5$, an input shaper with three or four impulses can also be designed because the sum of the corresponding impulse vectors can be made zero. Table 1 shows the SD shaper with five impulses and two input shapers with three and four impulses obtained using design procedure (ii) and (iii) except (i) in the previous section. But this input shaper with three or four impulses is not a satisfactory solution because of its high sensitivity to modeling errors compared to the SD shaper. Fig 8 shows that the SD shaper with five impulses for $t_{s}=0.85\text{ s}$ is less sensitive to modeling errors in $\omega_{n}$ than the input shaper with three or four impulses for $t_{s}=0.85\text{ s}$ since the *V* values of the SD shaper are smaller than the *V* values of two input shapers around $\omega_{n} / \hat{\omega}_{n}=1$ in Fig 8. Main reason for better robustness of SD shapers than input shapers with less impulses for the same shaping time is that SD shapers satisfy more derivative constraints than input shapers with less impulses.

**Fig 8. Sensitivity curves of the SD shaper and input shapers with three or four impulses for the case with the shaping time** $t_{s}\mathbf{=0.85}$**s for an underdamped system with** $\hat{\omega}_{n}\mathbf{=2 Hz}$ **and** $\zeta\mathbf{=0.1}$**.** The SD shaper with five impulses is more robust to modeling errors than the input shaper with three or four impulses for $t_{s}=0.85\text{ s}$.

**Table 1. The SD shaper and two input shapers with three and four impulses for the shaping time** $t_{s}\mathbf{=0.85}$**s.**

| **Input shapers** | **Shaping time** $t_{s}\mathbf{=0.85}$**s** |
| --- | --- |
| SD shaper with  five impulses | $\left[\begin{matrix} t_{i} \\ A_{i} \end{matrix}\right]=\left[\begin{matrix} 0, & 0.1143, & 0.3513 & 0.5998, & 0.85 \\ 0.0282, & 0.1859, & 0.4149, & 0.2989, & 0.0722 \end{matrix}\right]$ |
| Input shaper with  four impulses | $\left[\begin{matrix} t_{i} \\ A_{i} \end{matrix}\right]=\left[\begin{matrix} 0, & 0.3445, & 0.5979, & 0.85 \\ 0.0086, & 0.3341, & 0.4820, & 0.1753 \end{matrix}\right]$ |
| Input shaper with  three impulses | $\left[\begin{matrix} t_{i} \\ A_{i} \end{matrix}\right]=\left[\begin{matrix} 0, & 0.2473, & 0.85 \\ 0.5754, & 0.4143, & 0.0103 \end{matrix}\right]$ |

As I mentioned in my previous report, the procedure here seems somewhat “ad hoc,” which is not necessarily a bad thing. The idea of adding a time constraint and then trying to produce a reasonably “insensitive” input shaper by using several impulses and impose constraints on the input shaper is interesting. If the results are largely experimental, say so and provide tables and figures to compare your results to results attained using more standard methods. Show the reader that your design methods lead to practical input shapers that outperform the well known ones.

Answer: According to the reviewer’s comment, I added one more sentence about ad hoc procedure below Equation (7) as follows.

Design of an SD shaper (line 135): The correlations shown in (7) follow from observing impulse vector diagrams and ZVDn shapers [21].

[Further explanation to the reviewer] The main idea of the ZV to ZVD^n^ shapers is that, by adding more derivative constraints, the robustness to modeling errors increases and also the shaping time increases. If we study these shapers more deeply, it is obvious that we can increase the number of impulses in an input shaper to maximize the robustness to modeling errors as shaping time increases. Thus, I believe that the readers who are interested in input shaping control could understand why SD shapers require N impulses for different T_s_ with this description in the revised manuscript.

Since the manuscript proposed the input shaper that could control the shaping time for the first time, there are no other input shapers to compare, which can control the shaping times. Thus, the performance between SD shapers and well-known ZV and ZVD shapers are compared at Fig 4, 5, 6, and 7 using sensitivity curves and step responses.

Minor Issues

Why do you not try to add impulses even for smaller values of T_s? Would that not allow you to make the input shaper more “robust?”

Answer: If more impulses are added for smaller values of $T_{s}$, it may not be possible to obtain a solution. For example, if we try to find an SD shaper with four impulses for $0.5<T_{s}\leq1$, it is not possible to obtain a solution to satisfy all constraints in (24) because derivative constraints in (24) cannot be satisfied within a shaping time of one period.

On line 17, the word “are” should be replace by “is”.
Answer: ‘a specified-duration (SD) shaper and a systematic method’ is the subject of the sentence, and so ‘are’ is correct. This was double checked during copyediting process.

<Response during copyediting process>

On line 27, I would say that one often wants to reduce vibrations. Sometimes, one is actually trying to build an oscillator.
Answer: According to the reviewer’s comment, ‘vibrations should in general be suppressed rapidly to avoid system performance degradation’ was revised as follows.

In engineering systems, it is often beneficial to be able to rapidly reduce vibrations to avoid system performance degradation.

On line 35, you should say “Since the 1980s”. (You need to add the word “the.”) Also, please note that in your current bibliography, you have additional articles by some of the authors mentioned.

Answer: According to the reviewer’s comment, I added ‘the’, and added more references there.

On line 42, I would say “an input shaper is a linear time-invariant system how impulse response consists of…”.
Answer: I would prefer the current description which emphasizes the key characteristics of input shapers.

On line 80, I would use “performance” rather than “validity.”
Answer: According to the reviewer’s comment, the ‘validity’ was replaced with ‘performance’. Thank you.

On line 342, please replace “if actual” with “if the actual”.
Answer: According to the reviewer’s comment, ‘the’ is added on line 343. Thank you.

Please copyedit the article carefully before resubmitting. (This is almost always helpful.)

Answer: The manuscript was copyedited once again by Editage (www.editage.co.kr). Thank you for your valuable comments.

**Response to Reviewer #3**

Reviewer #3: The results described in the manuscript are predictable, but the paper could be useful for a reader interested in input shaping methods. Moreover, it is easy to understand and well written. The author has adequately addressed my comments, and I think this has been useful to improve the quality of the paper, which is now adequate for publication.

Answer: Thank you.

**Response to Reviewer #4**

Reviewer #4: The author addresses several of my comments and fail to show the novelty of the proposed shaper especially surveying the literature. I will assure the paper could be published if the author either extend his shaper to multi-mode system or even explain how can be extended. One way is by using convolution where the other can be based on solving a system of nonlinear equations. Hence, the author could address this issue by reviewing the following papers:

1. “An extension of command shaping methods for controlling residual vibration using frequency sampling” by N. C. Singer and W. P. Seering.
2. “Using input command pre-shaping to suppress multiple mode vibration” by J. M. Hyde and W. P. Seering.
3. “Design of input shapers using modal cost for multi-mode systems” by R. Kumar and T. Singh.
4. “Command Shaping for Sloshing Suppression of a Suspended Liquid Container” by A. Alshaya and K. Alghanim
5. “Robust multi-steps input command for liquid sloshing control” by A. Alshaya and A. Alshayji.
6. “Robust time-delay control of multimode systems” by T. Singh and S. R. Vadali.
7. “Shaping Container Motion for Multimode and Robust Slosh Suppression” by B. Pridgen, K. Bai, and W. Singhose.

Answer: Since the multi-mode shaper is a different issue in input shaping control, the author partially does not agree to the reviewer’s comment, and thus the above recommended references 1, 2, 4, 5 and 7 were additionally cited at the Introduction of the revised manuscript. The recommended reference 6 was already cited at the previous version of the manuscript.

The advantages of the proposed SD shaper is its capability to be convolved with other filtering techniques; notch, low pass, or even band-gap filters. The author can for example consult the papers for Jie Huang who extend the input shaper to make it smooth and robust. Please see the following:
1. “Command Generation for Flexible Systems by Input Shaping and Command Smoothing” by W. Singhose, R. Eloundou, J. Lawrence.
2. “Vibration reduction for flexible systems by command smoothing” by X. Xie, J. Huang, and Z. Liang.

Answer: According to the reviewer’s comment, the above recommended reference 2 was additionally cited at the Introduction of the revised manuscript.

It is quite important that the author cites the new relevant papers to promote his work and demonstrate its innovative and not outdated.
Again, the author can either discuss in general how a negative impulse can added to his proposed SD shaper and how the SD shaper can be extended to multi-mode system. A paragraph stating the idea is fine. However, I, personally, recommend a demonstrated example especially the author states that he left where the experimental apparatus is, and he (the author) has no access now. Discussing these issues and showing the further capability of the proposed shaper (extension to multi-mode) will make the paper to be above the others. It is worth mentioning that not only the objective of a research is just to publish it but also to make it readable and citable.

Answer: As I mentioned above, the multi-mode shaper and negative SD shaper are different issues with the present manuscript of the specified-duration shapers. Thus, the extension of the SD shapers to multi-mode shapers and negative shapers could be further research topic.

According to the reviewer’s comment, I have included a sentence at the end of the Conclusion as follows.

For further study, the SD shaper proposed in this paper could be extended to a negative SD shaper or a multi-mode SD shaper.

General Comments:
1. Add the overmentioned papers in the introduction starting from the second sentence “for example the forced lateral vibration … “.

Answer: According to the reviewer’s comment, I added most of the recommended references.

2. Add the paper entitled “An extension of command shaping methods for controlling residual vibration using frequency sampling” with references 17-19 since that paper presents EI shaper.

Answer: According to the reviewer’s comment, I added the recommended reference there.

3. Even though I disagree with the need of writing the damped natural frequency as a new equation, but if the author prefers doing that please add an equation number.

Answer: According to the other reviewer’s comment, I rewrote the definition of the damped natural frequency $\omega_{d}$ as a new line without Equation number because I did not cite that equation number in the text of the manuscript. However, according to your recommendation, I added Equation number at the definition of $\omega_{d}$.

By the way, the simple definition of $\omega_{d}$ could be a part of the text because all the readers of this paper possibly already know the definition of $\omega_{d}$ (originally I only wanted to inform the notation), or the definition of $\omega_{d}$ could be a new line without Equation number because I do not cite that equation number in the text of the revised manuscript.

4. I do not nee the author to re-produce the experimental results again. I only need the author to add the ZV and ZVD duration in the legends.

Answer: According to the reviewer’s comment, I added the ZV and ZVD duration in Fig 5A and 5B legends.

5. The same thing goes with the sensitivity curves.

Answer: According to the reviewer’s comment, I added the ZV and ZVD duration in the Fig 4 and 6 legends.
