# Supplementary material for: Specified-duration shapers for suppressing residual vibrations
Source: PLoS One. 2022 Nov 28;17(11):e0276669. doi: 10.1371/journal.pone.0276669 (PMC9704665; doi:10.1371/journal.pone.0276669)
Supplement: Attachment — Submitted filename: Response to Reviewers_3.docx [file pone.0276669.s003.docx]

**Response to Reviewer #1**

**Reviewer #1:**

I read the revised version of your paper with interest. I have several comments and suggestions. Number 7, below, is, in my opinion, very important as it provides a partial justification of your equation 7 -- which is central to your design procedure.

1. In several places (such as lines 16 and 75), you speak of “maximal robustness.” I think it would be better to speak of robustness without mentioning maximality. It is not clear that your controller is maximally robust.

Answer: Detailed design procedure of the SD shaper, for example, with three impulses is as follows.

(1) Specify a shaping time $t_{s}$ between a half period and one period.
(2) Assume $A_{3}=0.01$. Then find the best $A_{1}, A_{2}, t_{2}$ satisfying Eq. (8)-(10) and obtain insensitivity $I_{0.05}$ (that is a robustness measure to modeling errors). [‘the best’ in view of the errors in Eq. (8)-(10)].
(3) Let $A_{3}=0.02$. Then find the best $A_{1}, A_{2}, t_{2}$ satisfying Eq. (8)-(10) and obtain insensitivity.
(4) Let $A_{3}=0.03$. Then find the best $A_{1}, A_{2}, t_{2}$ satisfying Eq. (8)-(10) and obtain insensitivity.

(5) Let $A_{3}=0.99$. Then find the best $A_{1}, A_{2}, t_{2}$ satisfying Eq. (8)-(10) and obtain insensitivity.
(6) Find the biggest $I_{0.05}$ among $I_{0.05}$s obtained (1)-(5).
(7) Then $A_{1}, A_{2}, A_{3}, t_{2}, t_{3}(=t_{s})$ with the biggest $I_{0.05}$ is the SD shaper for given $t_{s}$.

Therefore, in this viewpoint, the SD shaper is the input shaper with maximal robustness (i.e., biggest $I_{0.05}$) for given $t_{s}$.

Thus, I would prefer to keep the expression ‘maximal robustness’ in the manuscript. However, according to the reviewer’s comment, I added ‘in view of 5% insensitivity’ as follows.

[line 74] In this paper, a novel specified-duration (SD) shaper that satisfies an arbitrarily specified shaping time with maximal robustness to modeling errors in view of 5% insensitivity is proposed.

[line 523] This paper introduced an SD shaper that can suppress residual vibrations at an arbitrarily specified shaping time with maximal robustness to modeling errors in view of 5% insensitivity.

2. In line 37, the citation [15-20] contains a paper not authored by Singer, Singhose, Singh, and it is missing some papers written by one or more of them that appear in your bibliography.

Answer: Thank you for the detailed comment. According to the reviewer’s comment, I have revised the manuscript as follows.

[line 36] Since the 1980s, N. Singer, J. Hyde, W. Singhose, and T. Singh have considerably improved input-shaping control by adding derivative constraints [15-19].

[line 44] The convolution of this impulse sequence (i.e., input shaper) with the command input of the system can remove residual vibration caused by the command input [20].

3. Before line 128, I think that you should explain that you need some flexibility in order to achieve robustness. In order to give yourself the flexibility you need, you add impulses. Then you can discuss how you decided how many impulses to add.

Answer: According to the reviewer’s comment, I added a paragraph explaining the thinking logic.

[line 128] It is well known that the ZV, ZVD and ZVD*^n^* shapers have the shaping times of a half period, one period, and $(n+1)/2$period, which are $T_{s}=0.5$, $T_{s}=1$, and $T_{s}=(n+1)/2$, respectively [21]. Also, it is well known that the residual vibration is removed if the resultant of the impulse vectors in an impulse vector diagram is zero [21]. Observing these facts, the rule of deciding the number of impulses of SD shapers are determined as follows.

If the dimensionless shaping time $T_{s}$ is between 0.5 and 1 (that is, between half and a period), the SD shaper consists of three impulses. …

4. In line 135, I think that I would replace “correlation” with “correspondence.”

Answer: I removed this line because the content of this line is described in detail in line 128-132 as above.

5. In line 149, I would replace “five impulses are required” with “(7) states that we should use five impulses”.

Answer: According to the reviewer’s comment, I replaced “five impulses are required” with “Eq (7) states that we should use five impulses”. Thank you for the valuable comment.

6. After line 152, refer the reader to the material in lines 406-424 where s/he will see a partial justification for using 5 impulses.

Answer: According to the reviewer’s comment, I added a sentence in line 157 as follows.

[line 157] For an exemplified justification for using five impulses in this case, refer to the last paragraph of the next section, Analysis of SD shapers.

7. In lines 406-424, you added a very informative example. I would like to see one additional case there – an input shaper with six impulses. So far you have justified using more than three or four impulses. It is important to show that six is not a major improvement over five.

Answer: According to the reviewer’s comment, I added an input shaper with six impulses and compared with the SD shaper with five impulses. Specifically, the paragraph starting at line 415 and Fig. 8 and Table 1 are revised as follows.

If $t_{s}=0.85\text{ s}$ for the underdamped system with $\hat{\omega}_{n}=2\mathbf{ }\text{Hz}$ and $\zeta=0.1$, the dimensionless shaping time is $T_{s}\approx1.692>1.5$, and thus, the SD shaper is composed of five impulses according to (7). However, considering the impulse vector diagram for the case with $T_{s}>1.5$, an input shaper with three, four or six impulses can also be designed because the sum of the corresponding impulse vectors can be made to be zero. Table 1 shows the SD shaper with five impulses and three input shapers with three, four and six impulses obtained using design procedures (ii) and (iii) and excluding procedure (i) in the previous section. However, this input shaper with three, four or six impulses is not a satisfactory solution because of its high sensitivity to modeling errors compared to the SD shaper. Fig 8 shows that the SD shaper with five impulses for $t_{s}=0.85\text{ s}$ is less sensitive to modeling errors than the input shaper with three, four or six impulses for $t_{s}=0.85\text{ s}$ since the *V* values of the SD shaper are smaller than the *V* values of the three input shapers around $\omega_{n} / \hat{\omega}_{n}=1$ in Fig 8. The main reason for the improved robustness of the SD shapers than input shapers with less impulses for the same shaping time is that SD shapers satisfy more derivative constraints than input shapers with less impulses. However, it may not be possible to obtain an input shaper with six impulses because all nine constraints [Eq (26) plus two more third-order derivative constraints] cannot be satisfied for the case with $t_{s}=0.85\text{ s}$. Even if we find an input shaper with six impulses for $t_{s}=0.85\text{ s}$ numerically, it may be an approximate solution with less robustness such that the sum of $A_{i}$ is close to one (not exact one) as can be checked in the last input shaper of Table 1. Note that the sum of $A_{i}$ of the first three input shapers in Table 1 are exactly one, but the sum of $A_{i}$ of the last input shaper is 1.0024.

**Fig 8. Sensitivity curves of the SD shaper with five impulses and input shapers with three, four or six impulses for the case with the shaping time** $t_{s}\mathbf{=0.85}$**s for an underdamped system with** $\hat{\omega}_{n}\mathbf{=2 Hz}$ **and** $\zeta\mathbf{=0.1}$**.** The SD shaper with five impulses is more robust to modeling errors than the input shaper with three, four or six impulses for $t_{s}=0.85\text{ s}$.

**Table 1. The SD shaper with five impulses and input shapers with three, four and six impulses for the shaping time** $t_{s}\mathbf{=0.85}$**s.**

| **Input shapers** | **Shaping time** $t_{s}\mathbf{=0.85}$**s** |
| --- | --- |
| SD shaper with  five impulses | $\left[\begin{matrix} t_{i} \\ A_{i} \end{matrix}\right]=\left[\begin{matrix} 0, & 0.2273, & 0.4346 & 0.6335, & 0.85 \\ 0.1436, & 0.3301, & 0.3060, & 0.1756, & 0.0447 \end{matrix}\right]$ |
| Input shaper with four impulses | $\left[\begin{matrix} t_{i} \\ A_{i} \end{matrix}\right]=\left[\begin{matrix} 0, & 0.2838, & 0.5715, & 0.85 \\ 0.2198, & 0.3993, & 0.2950, & 0.0859 \end{matrix}\right]$ |
| Input shaper with three impulses | $\left[\begin{matrix} t_{i} \\ A_{i} \end{matrix}\right]=\left[\begin{matrix} 0, & 0.2473, & 0.85 \\ 0.5754, & 0.4143, & 0.0103 \end{matrix}\right]$ |
| Input shaper with six impulses | $\left[\begin{matrix} t_{i} \\ A_{i} \end{matrix}\right]=\left[\begin{matrix} 0, & 0.2182, & 0.3514, & 0.3532, & 0.6001, & 0.85 \\ 0.0547, & 0.0534, & 0.0274, & 0.3272, & 0.4160, & 0.1237 \end{matrix}\right]$ |

Apology: During the justification process, I found a typo in the MATLAB simulation code and corrected it. Thus, the numeric values of Eq. (25), (27) and a part of Table 1 are changed, and accordingly Fig. 6, Fig. 7 and Fig. 8 are redrawn.

However, there is no change in the technical facts and context of the manuscript.

Thank you for valuable comments.

Prof. Brian Byunghyun Kang
