# Supplementary material for: Specified-duration shapers for suppressing residual vibrations
Source: PLoS One. 2022 Nov 28;17(11):e0276669. doi: 10.1371/journal.pone.0276669 (PMC9704665; doi:10.1371/journal.pone.0276669)
Supplement: Attachment — Submitted filename: Response to Reviewers_4.docx [file pone.0276669.s004.docx]

**Response to Reviewer #1**

**Reviewer #1:**

I read your revisions with interest. In what follows, the numbering is the same as the numbering in my previous report. As is implied in 1 and 7, I think that “maximal robustness” must be defined clearly.

1. When you speak of maximal robustness, you seem to mean relative to this procedure when a free parameter is added. Generally speaking, maximal robustness would be expected to mean relative to all choices of all relevant parameters.

Answer: I accept your criticism, and I agree to your opinion on the term ‘maximal robustness’. My SD shaper has just the biggest 5% insensitivity among all the cases with different A_N_ values (by increasing A_N_ by 0.01 from 0.01 to 0.99). In this way, only discrete values of A_N_ with the interval 0.01 from 0.01 to 0.99 are considered in this paper. Thus, I revised “with maximal robustness to modeling errors” to “with the biggest 5% insensitivity to modeling errors for a free parameter of the last impulse magnitude” in all the manuscript. Specifically, I revised the manuscript as follows.

[line 72] “In this paper, a novel specified-duration (SD) shaper that satisfies an arbitrarily specified shaping time with maximal robustness to modeling errors is proposed.” → “In this paper, a novel specified-duration (SD) shaper that satisfies an arbitrarily specified shaping time with the biggest 5% insensitivity to modeling errors for a free parameter of the last impulse magnitude is proposed.”

[line 159] “maximize the robustness to modeling errors” → “maximize the 5% insensitivity by increasing $A_{N}$ discretely by 0.01 from 0.01 to 0.99”

[line 166] “$A_{N}$ is a free parameter to maximize the robustness to modeling errors” → “$A_{N}$ is a free parameter (discrete values with 0.01 intervals) to maximize the 5% insensitivity to modeling errors”

[line 185] “the robustness to modeling errors” → “the 5% insensitivity to modeling errors”

[line 520] “an SD shaper that can suppress residual vibrations at an arbitrarily specified shaping time with maximal robustness to modeling errors in view of 5% insensitivity.” → “an SD shaper that can suppress residual vibrations at an arbitrarily specified shaping time with the biggest 5% insensitivity to modeling errors for a free parameter of the last impulse magnitude.”

2. [21 – 26] also include Singer and Singhose among their authors.

Answer: According to your last review comment, I added ‘J. Hyde’ in the text of the last revision because he was another first author of the cited papers, and in line 36, I wrote “Since the 1980s, N. Singer, J. Hyde, W. Singhose, and T. Singh have considerably improved input-shaping control by adding derivative constraints [15-19].” The references [15-19] insist that by adding derivative constraints, the robustness to modeling errors of the input shaper was improved. However, the main points of references [22-26] are not “increasing robustness by adding derivative constraints” but “increasing robustness by other ways, e.g., by using frequency sampling”. Thus, references [21], [22-25],[26] are cited at other places of the manuscript.

3. In my opinion, it is not clear from the text that you are adding impulses in order to add free parameters with which to optimize the design. I still think that this should be pointed out.

Answer: According to the reviewer’s comment, I added the following sentence in line 129.

Moreover, we need some flexibility in order to achieve robustness to modeling errors of the SD shaper, which can be accomplished by using a free parameter of the added last impulse.

Thank you for this comment to improve the readability of the manuscript.

4. Thank you.

5. Thank you.

6. Please reword the addition. I recommend, “For an example that provides a partial justification for using…”.

Answer: According to the reviewer’s good comment, I replaced “For an exemplified justification for using…” with “For an example that provides a partial justification for using…”. Thank you for recommending better English.

7. I think there is still work to be done. When you add degrees of freedom and optimize, the results should not get worse. If necessary, the optimization should return one amplitude that is set to zero.
I think that we are back to the question of what “maximal robustness” means. If you maximize over all parameter values, adding a new impulse cannot make things worse because forcing one impulse’s amplitude to zero brings us back to the “preceding case.”
If I understand what you are doing correctly, you should be able to fix t_6 – the time of the last pulse – and then optimize by choosing values for A_5,A_6, and t_5 and using the constraints for the five-impulse case to determine the rest of the coefficients. This is still not a search over all possible reasonable value of all the coefficients – so I still would not say that the solution is maximally robust, but it should make sure that your results are not worse than the case with five impulses – as when A_5 is set to zero and t_5 is greater than the value of t_4 in the SD shaper, you should recover the SD shaper. That is, my suggestion is to iterate over possible values of A_5,A_6, and t_5 and use the constraints from the 5-impulse (SD shaper) case to choose the rest of the coefficients.

Answer: There is the best number of impulses for the SD shaper according to the interval of the *specified shaping time*, which is Eq. (7). For a given specific shaping time, the number of impulses *N* is determined by Eq. (7), and so in this case, even if we add more impulses, the performance could not be improved when we follow the design procedure of the SD shaper in this manuscript.

I agree to your opinion on ‘not maximally robust’ or ‘not optimal’ because the SD shaper was found by comparing 5% insensitivity *I*_0.05_ for the cases with 0.01 intervals of *A*_N_ values. For this reason, I did not use the word ‘optimization’ or ‘optimal’ in the paper. Also, in the present manuscript according to the reviewer’s comment, I rephrased ‘maximal robustness’ with ‘the biggest 5% insensitivity to modeling errors for a free parameter of the last impulse magnitude’.

In the example of Fig. 8 and Table 1 in the paper, the SD shaper with five impulses for $t_{s}=0.85$s (therefore $1.5<T_{s}\leq2$) was obtained by seeking an input shaper with the biggest 5% insensitivity $I_{0.05}$ for a free parameter $A_{5}$ of the last impulse using *seven* constraints, in which $A_{5}$ was increased by 0.01 discretely from 0.01 to 0.99. Thus, the SD shaper for $t_{s}=0.85$s is not optimal mathematically for *continuous* variable $A_{5}$ (we are using discrete values of $A_{5}$ with 0.01 intervals) , and the input shaper with six impulses obtained from *nine* constraints cannot be reduced to SD shaper with five impulses (i.e., cannot be recovered to the SD shaper) even if we follow the procedure suggested by the reviewer. There are two reasons. First, the SD shaper with five impulses and the input shaper with six impulses are obtained from different sets of constraints (7 constraints and 9 constraints, respectively), and secondly, we are seeking input shapers numerically using fsolve() function with trust-region-reflective algorithm in MATLAB for *discrete values* of $A_{5}$ [from 0.01 (not 0) to 0.99 (not 1) with 0.01 intervals] for the SD shaper with five impulses, and for *discrete values* of $A_{6}$ [from 0.01 (not 0) to 0.99 (not 1) with 0.01 intervals] for the input shaper with six impulses. Even if we start from zero initial value in the iteration process, the input shaper with six impulses cannot be reduced to the SD shaper with five impulses by the above reason.

However, if we try to seek an input shaper with six impulses for $t_{s}=0.85$s using the SD shaper constraints (i.e., using *seven* constraints, not *nine* constraints, which is not the design procedure of the SD shaper in this paper), the input shaper with six impulses is approaching to the SD shaper under the condition of $A_{6}=0$ as commented by the reviewer, which is displayed in Answer_Figure 1 and Answer_Figure 2. It seems that the small difference between [these two figures] and [Fig. 8 and Table 1] comes from approximate numerical solutions by MATLAB. When trying to find a numerical solution for the input shaper with six impulses using seven constraints, we have to use levenberg-marquardt algorithm instead of trust-region-reflective algorithm in MATLAB because the number of unknowns and the number of equations are different (9 unknowns and 7 equations). If the number of unknowns and the number of equations are different, the trust-region-reflective algorithm does not work.

**Answer_Figure 1**. Screen capture of the MATLAB results for the input shaper with six impulses using *seven* constraints instead of *nine* constraints when $A_{6}=0$.

**Answer_Figure 2.** Sensitivity curves for the input shaper with six impulses using *seven* constraints instead of *nine* constraints when $A_{6}=0$. Sensitivity curves of the SD shaper with five impulses and the input shaper with six impulses are similar (but not exact). Compare this figure with Fig. 8 in the manuscript. It seems that the small difference between the SD shaper and the input shaper with 6 impulses comes from approximate numerical solutions obtained using fsolve() function in MATLAB.

Finally again, the main contribution of this paper is to design an input shaper *with an arbitrarily specified shaping time* systematically.

I appreciate your valuable comments, criticism and recommendations to improve the quality and readability of the manuscript. Thank you.

For reference to reproduce the simulation results of the paper, I attached the MATLAB screen captures for seeking the SD shaper in Fig. 8 and Table 1. For Fig. 8 and Table 1, I used the following options and initial values for fsolve() function in MATLAB.

% Assign option parameter values for fsolve() function

options.TolX = 1e-10;

options.MaxFunEvals = 5000;

options.MaxIter = 4000;

options.Algorithm = 'trust-region-reflective';

% Initial guess of input shaper with 3 impulses

x0 = [0.4 0.2 0.15*Td]; % [A1,A2,t2]

% Initial guess of input shaper with 4 impulses

x0 = [0.1, 0.2, 0.3, 0.3*Td 1*Td]; %[A1,A2,A3,t2,t3]

% Initial guess of SD shaper with 5 impulses

x0 = [0.1 0.2 0.2 0.2, 0.4*Td 0.8*Td 1.3*Td]; %[A1,A2,A3,A4,t2,t3,t4]

% Initial guess of input shaper with 6 impulses

x0 = [0.1 0.2 0.2 0.2 0.2, 0.4*Td 0.7*Td 1*Td 1.3*Td]; %[A1,A2,A3,A4,A5,t2,t3,t4,t5]

**Answer_Figure 3.** The screen capture of MATLAB results for $t_{s}=0.85$(Fig. 8 and Table 1)

If $t_{s}=0.8$s with the same initial conditions as above, the MATLAB code couldn’t find any solution for the input shaper with six impulses.

**Answer_Figure 4**. The screen capture of MATLAB results for $t_{s}=0.8$s. The solution for the input shaper with six impulses for $t_{s}=0.8$s could not be found.

**Answer_Figure 5**. The screen capture of MATLAB results for $t_{s}=0.8$ when using English version of MATLAB. This is for confirmation of computation and for reading English messages.

However, in this case with $t_{s}=0.8$s, it seemed that the input shapers with 3 or 4 impulses and SD shaper with 5 impulses could be computed because, in my MATLAB program, I sought input shapers with 3, 4, 5 and 6 impulses sequentially using different sets of constraints, and then displayed them in the monitor.

So when I checked the input shapers with 3 or 4 impulses and SD shaper with 5 impulses, they were as follows.

**Answer_Figure 6**. The screen capture of MATLAB results for $t_{s}=0.8$s. The solutions for the SD shaper with five impulses and input shapers with three and four impulses for $t_{s}=0.8$s can be obtained, but the input shaper with six impulses for $t_{s}=0.8$s could not be found.

**Answer_Figure 7**. The screen capture of MATLAB results for $t_{s}=0.8$s when using English version of MATLAB. This is for confirmation of computations and for reading English messages.
